# Author Correction: Using mortuary and burial data to place COVID-19 in Lusaka, Zambia within a global context

**DOI:** 10.1038/s41467-024-44940-w

**Published:** 2024-03-12

**Authors:** Richard J. Sheppard, Oliver J. Watson, Rachel Pieciak, James Lungu, Geoffrey Kwenda, Crispin Moyo, Stephen Longa Chanda, Gregory Barnsley, Nicholas F. Brazeau, Ines C. G. Gerard-Ursin, Daniela Olivera Mesa, Charles Whittaker, Simon Gregson, Lucy C. Okell, Azra C. Ghani, William B. MacLeod, Emanuele Del Fava, Alessia Melegaro, Jonas Z. Hines, Lloyd B. Mulenga, Patrick G. T. Walker, Lawrence Mwananyanda, Christopher J. Gill

**Affiliations:** 1 MRC Centre for Global Infectious Disease Analysis, Department of Infectious Disease Epidemiology, Imperial College, London, UK; 2 Department of Infectious Disease Epidemiology, Faculty of Epidemiology and Population Health, London School of Hygiene and Tropical Medicine, London, UK; 3 Department of Global Health, Boston University School of Public Health, Boston, MA USA; 4 Avencion Limited, Lusaka, Zambia; 5 Department of Biomedical Sciences, School of Health Sciences, University of Zambia, Lusaka, Zambia; 6 Zambia National Public Health Institute, Lusaka, Zambia; 7 Manicaland Centre for Public Health Research, Biomedical Research and Training Institute, Harare, Zimbabwe; 8 Carlo F. Dondena Centre for Research on Social Dynamics and Public Policy, Bocconi University, Milan, Italy; 9 Max Planck Institute for Demographic Research, Rostock, Germany; 10 Department of Social and Political Science, Bocconi University, Milano, Italy; 11 Centers for Disease Control and Prevention, Lusaka, Zambia; 12 Zambia Ministry of Health, Lusaka, Zambia

**Keywords:** Viral infection, Health policy, Epidemiology, SARS-CoV-2

Correction to: *Nature Communications* 10.1038/s41467-023-39288-6, published online 29 June 2023

In this Article, the affiliation details for Emanuele Del Fava & Alessia Melegaro were incorrectly given as ‘Carlo F. Dondena Centre for Research on Social Dynamics and Public Policy, Boccini University, Milan, Italy’ but should have been ‘Carlo F. Dondena Centre for Research on Social Dynamics and Public Policy, Bocconi University, Milan, Italy’. This has now been corrected in the PDF and HTML versions of the Article.

