# Supplementary material for: Using mortuary and burial data to place COVID-19 in Lusaka, Zambia within a global context
Source: Nat Commun. 2023 Jun 29;14:3840. doi: 10.1038/s41467-023-39288-6 (PMC10307769; doi:10.1038/s41467-023-39288-6)
Supplement: Supplementary file 1 — Supplementary Information [file 41467_2023_39288_MOESM1_ESM.pdf]

Supplementary Information:

Using mortuary and burial data to place COVID-19 in Lusaka, Zambia within a global context

Richard J. Sheppard[1], Oliver J. Watson[1,2], Rachel Pieciak[3], James Lungu[4], Geoffrey Kwenda[5], Crispin Moyo[4], Stephen Longa Chanda[6], Gregory Barnsley[1], Nicholas F. Brazeau[1], Ines C. G. Gerard-Ursin[1], Daniela Olivera Mesa[1], Charles Whittaker[1], Simon Gregson[1,7], Lucy C. Okell[1], Azra C. Ghani[1], William B. MacLeod[3], Emanuele Del Fava[8,9], Alessia Melegaro[8,10], Jonas Z. Hines[11], Lloyd B. Mulenga[12], Patrick G. T. Walker[1*†], Lawrence Mwananyanda[3,4]*, Christopher J. Gill[3]*

[1]MRC Centre for Global Infectious Disease Analysis, Department of Infectious Disease Epidemiology, Imperial College, London.

[2]Department of Infectious Disease Epidemiology, Faculty of Epidemiology and Population Health, London School of Hygiene and Tropical Medicine, London, UK.

[3]Department of Global Health, Boston University School of Public Health, Boston, MA 02118, USA.

[4]Avencion Limited, Lusaka, Zambia.

[5]Department of Biomedical Sciences, School of Health Sciences, University of Zambia, Lusaka, Zambia

[6]Zambia National Public Health Institute

[7]Manicaland Centre for Public Health Research, Biomedical Research and Training Institute, Harare, Zimbabwe

[8]Carlo F. Dondena Centre for Research on Social Dynamics and Public Policy, Boccini University, Milan, Italy.

[9]Max Planck Institute for Demographic Research, Rostock, Germany.

[10]Department of Social and Political Science, Bocconi University, Milano, Italy.

[11]Centers for Disease Control and Prevention, Lusaka, Zambia.

[12]Zambia Ministry of Health, Lusaka Zambia.

* Contributed Equally.

† Corresponding Author.

For the purpose of open access, the author has applied a 'Creative Commons Attribution (CC BY) licence (where permitted by UKRI, 'Open Government Licence' or 'Creative Commons Attribution No-derivatives (CC-BY-ND) licence' may be stated instead) to any Author Accepted Manuscript version arising.

Supplementary Figures

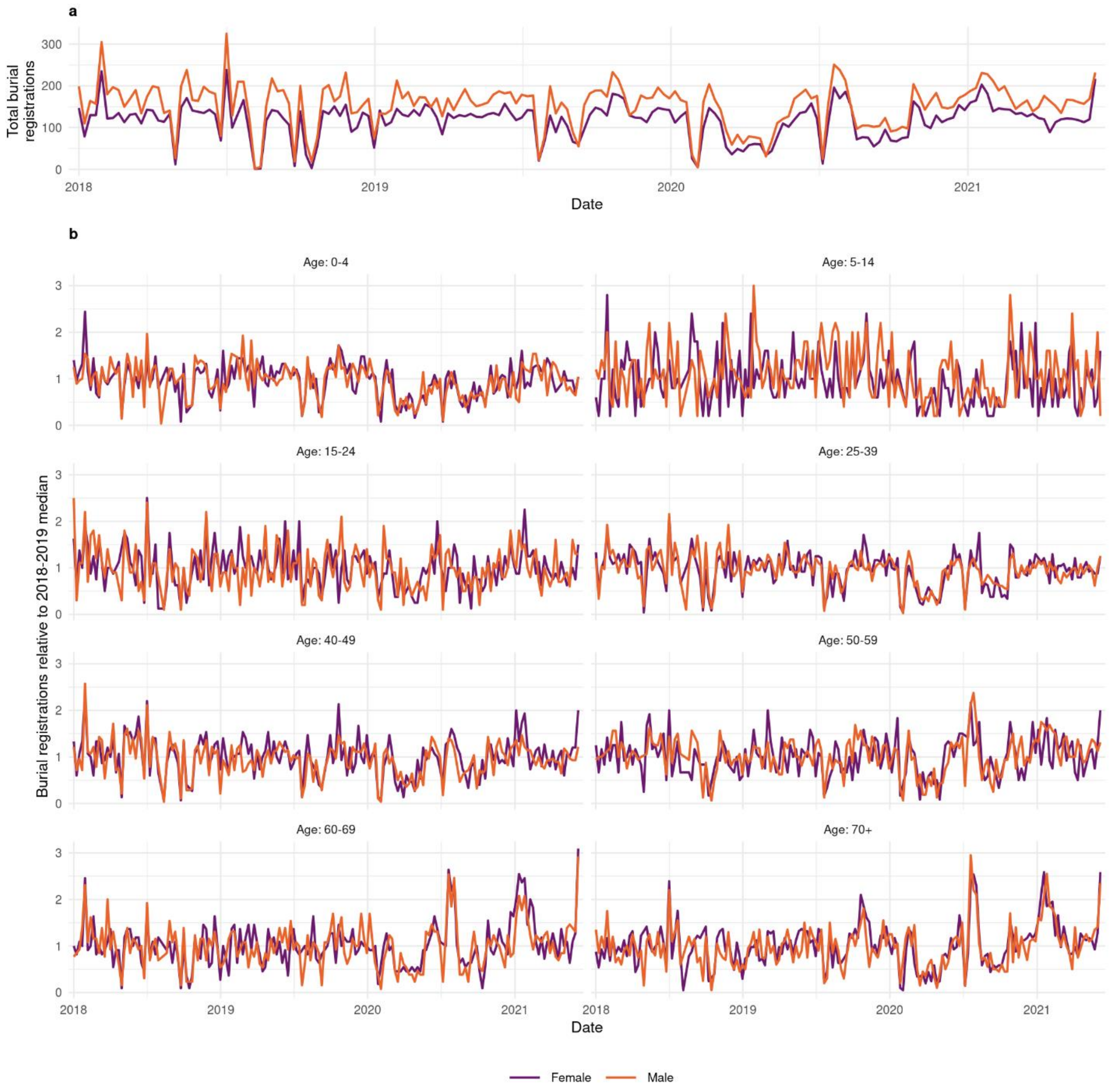

Supplementary Figure 1|Sex disaggregated burial registrations. Panel (a) shows the total weekly burial registrations through time, plotted by sex. Panel (b) shows the weekly deaths by age group and sex, relative to 2018-2019 medians.

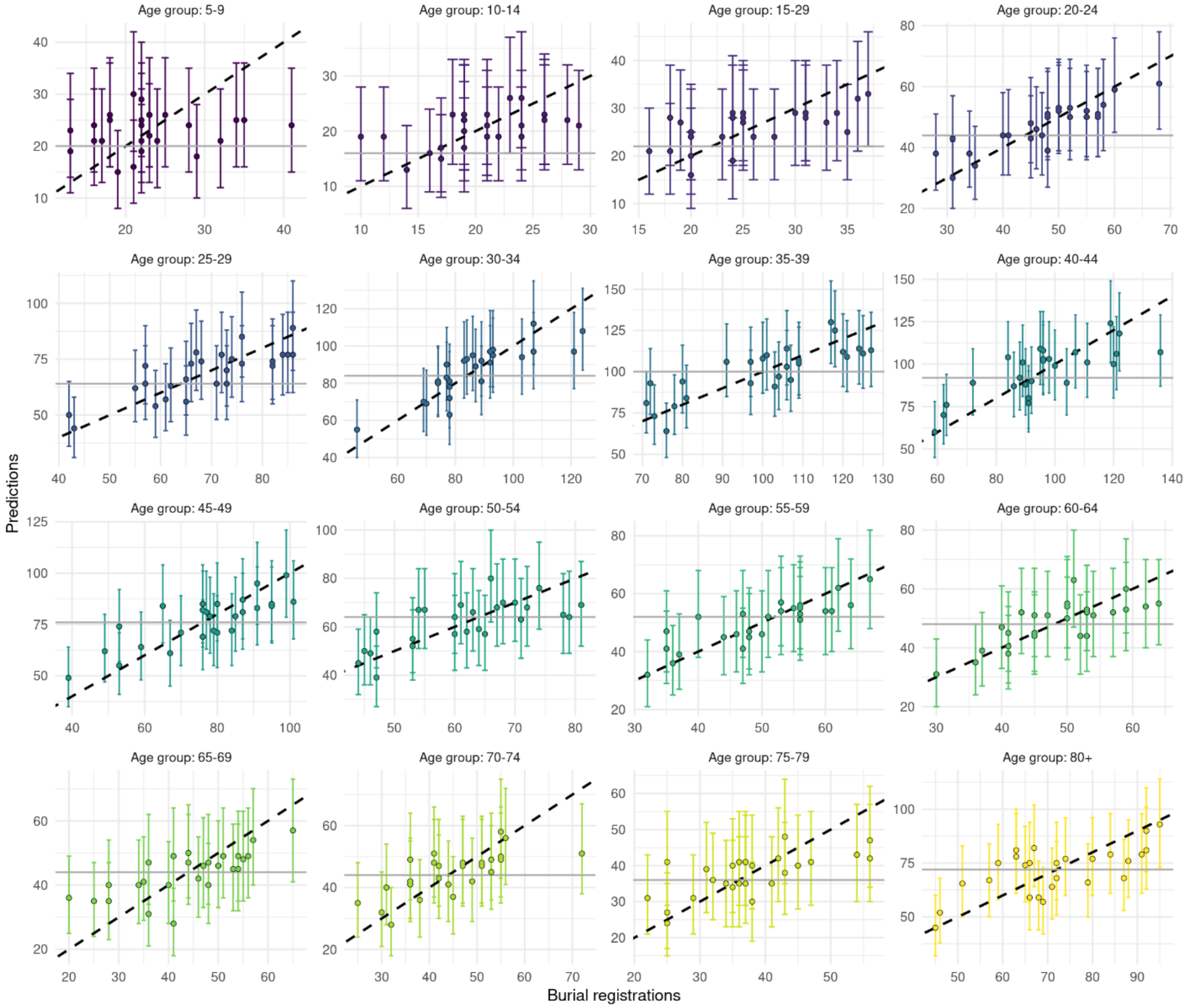

Supplementary Figure 2|Burial age structure k-fold cross validation. Comparison of excess burial registration predictions k-fold cross validation with 2018-2019 median (grey line). Points with bars show median with 95% credible intervals from 12,500 d.

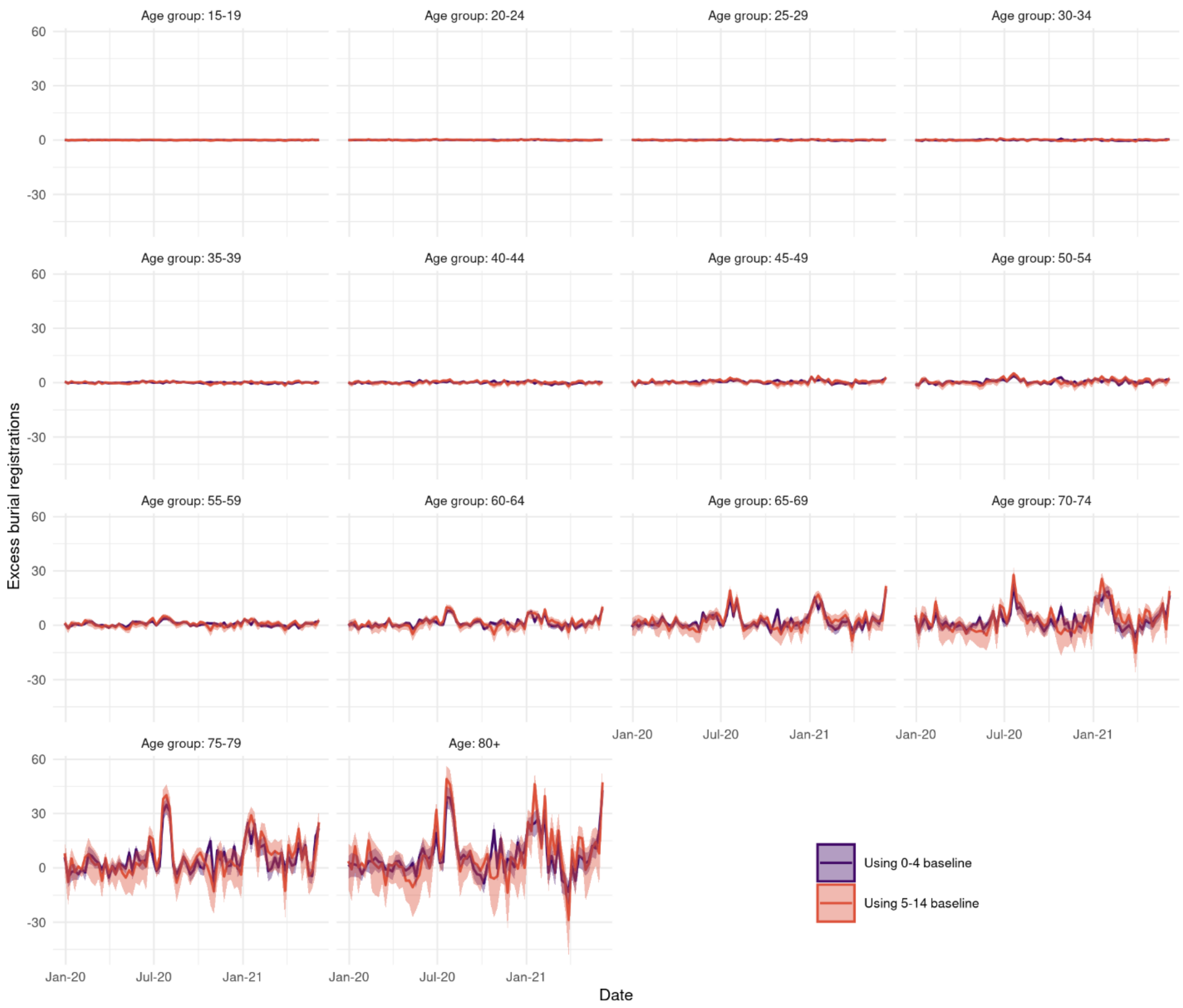

Supplementary Figure 3|Excess burial registration comparison. Comparison of excess burial registration estimates using a 0-4 age group baseline with estimates using a 5-14 age group baseline. Lines and ribbons show medians with 95% credible intervals. See Methods for more details.

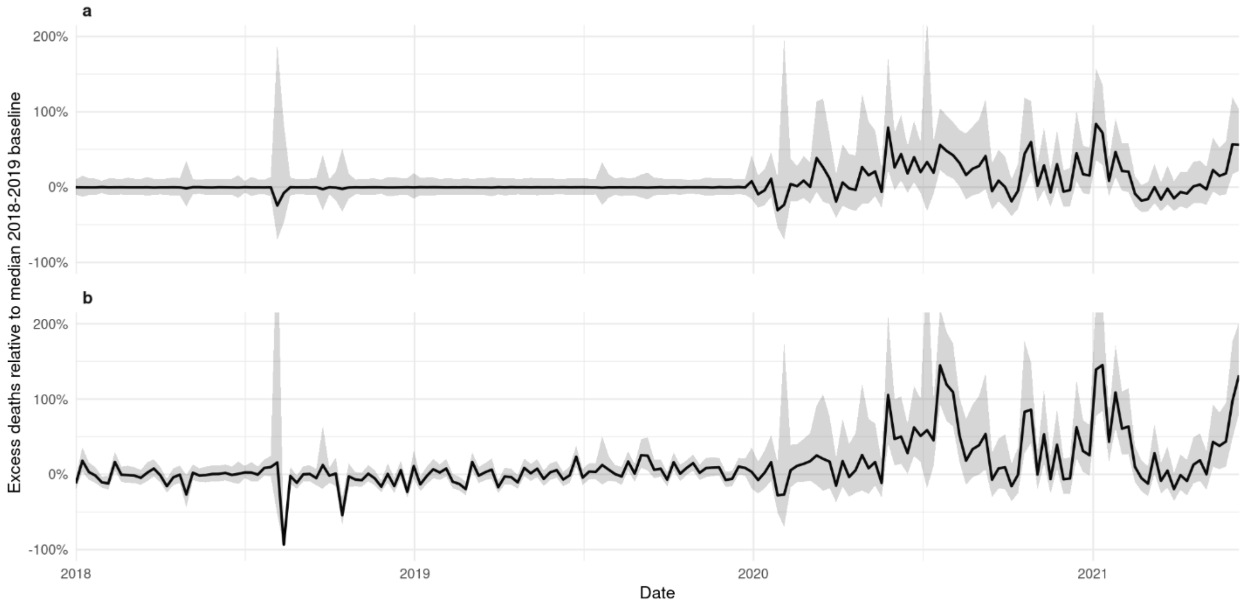

Supplementary Figure 4|Excess mortality relative to 2018-2019 median. Patterns shown in (a) all ages and (b) 50+ age group. Lines and ribbons show medians with 95% credible intervals.

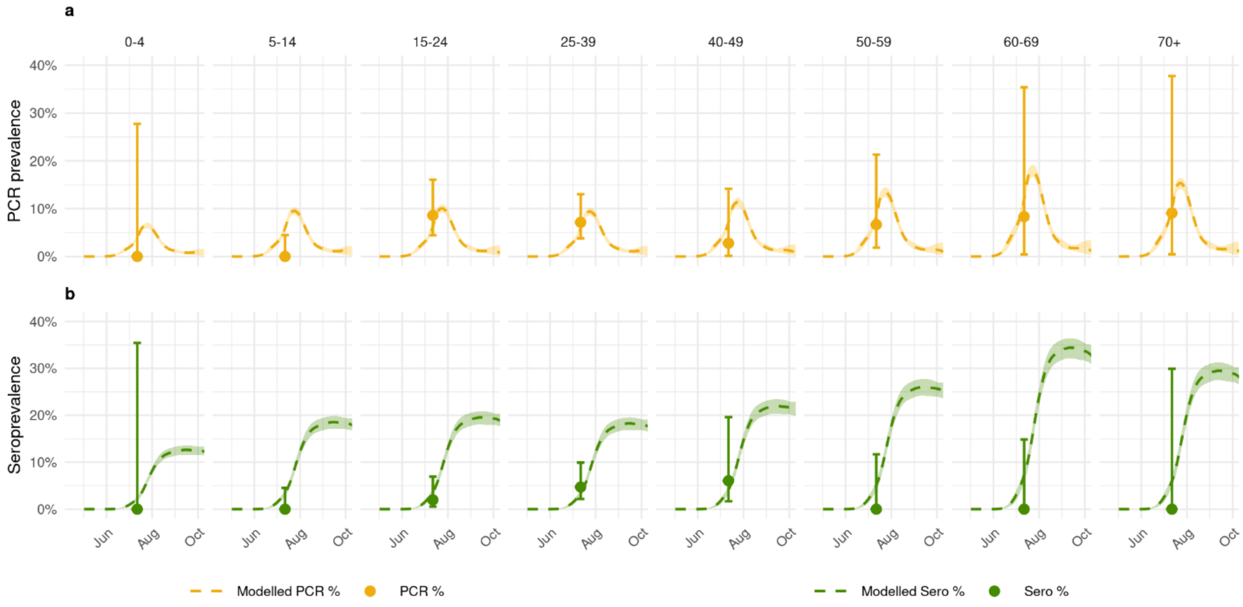

Supplementary Figure 5|SAR-CoV-2 polymerase chain reaction (PCR) prevalence and seroprevalence (Sero) by age group during 2020. Points and bars show mean and 95% binomial confidence intervals for (a) PCR prevalence (n=10, 82, 93, 126, 36, 30, 12 and 11 for each age group, left to right) or (b) seroprevalence data (n=7, 81, 101, 127, 33, 29, 22 and 9 for each age group, left to right). Dotted lines and ribbons show median and 95% credible intervals of model fit.

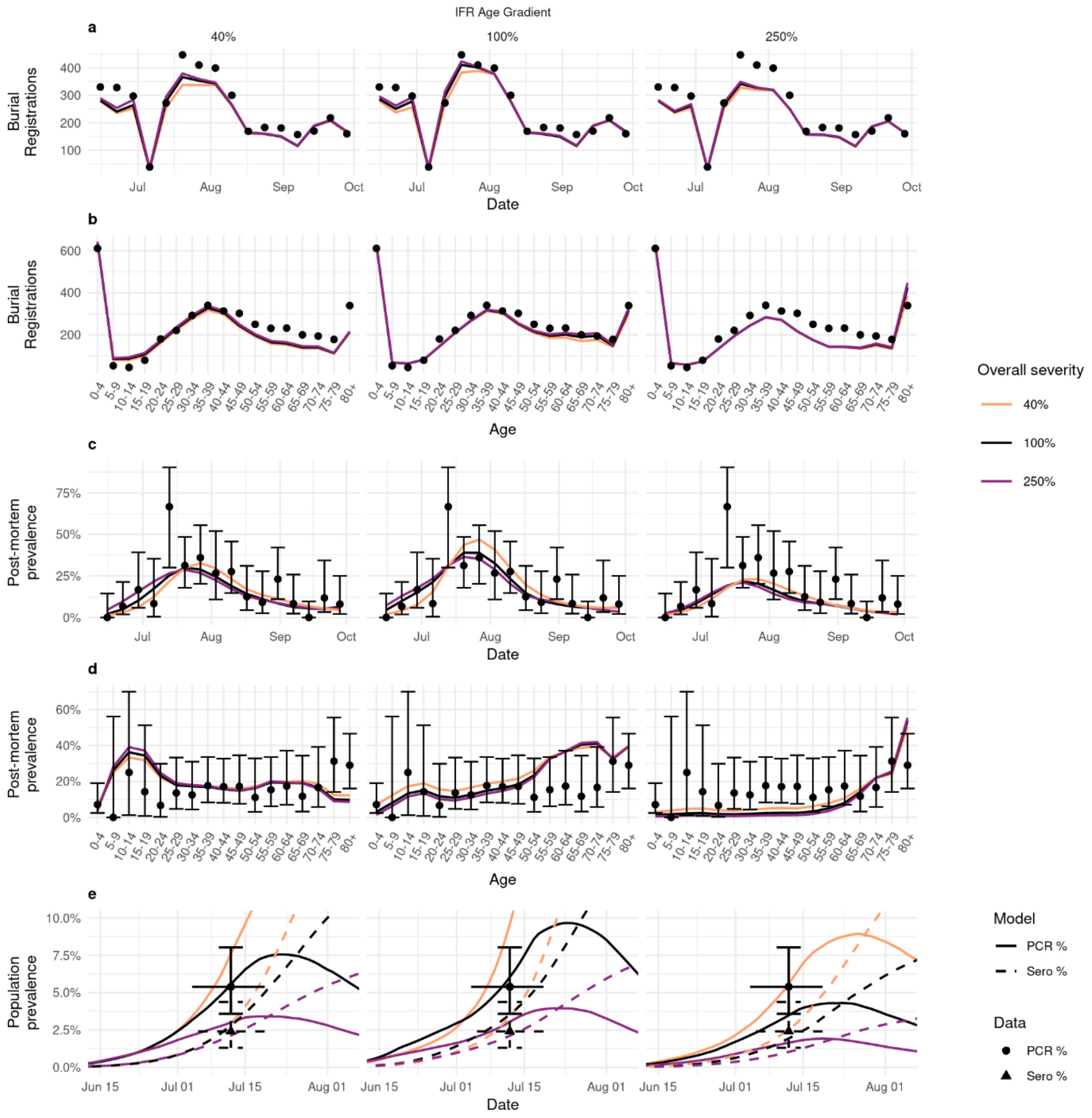

Supplementary Figure 6|Model fits varying age-gradient and overall infection-fatality ratio (IFR) assumptions. Facets show model fits as age-gradient of IFR varies (0.4, 1 and 2.5 times the default assumptions) while colours show fits as overall IFR varies (0.4, 1 and 2.5 times the default assumptions) for a given age-gradient. All plots show the median of 100 model replicates fit to the data. (a-b) show the model fit to burial registrations by age and week. (c-d) show the model fit to total (causal and coincidental COVID-19) post-mortem polymerase chain reaction (PCR) positivity, with 95% binomial confidence intervals. (e) shows fit to population PCR prevalence and seroprevalence (Sero), with 95% binomial confidence intervals given and the time frame of data sampling given by horizontal error bars.

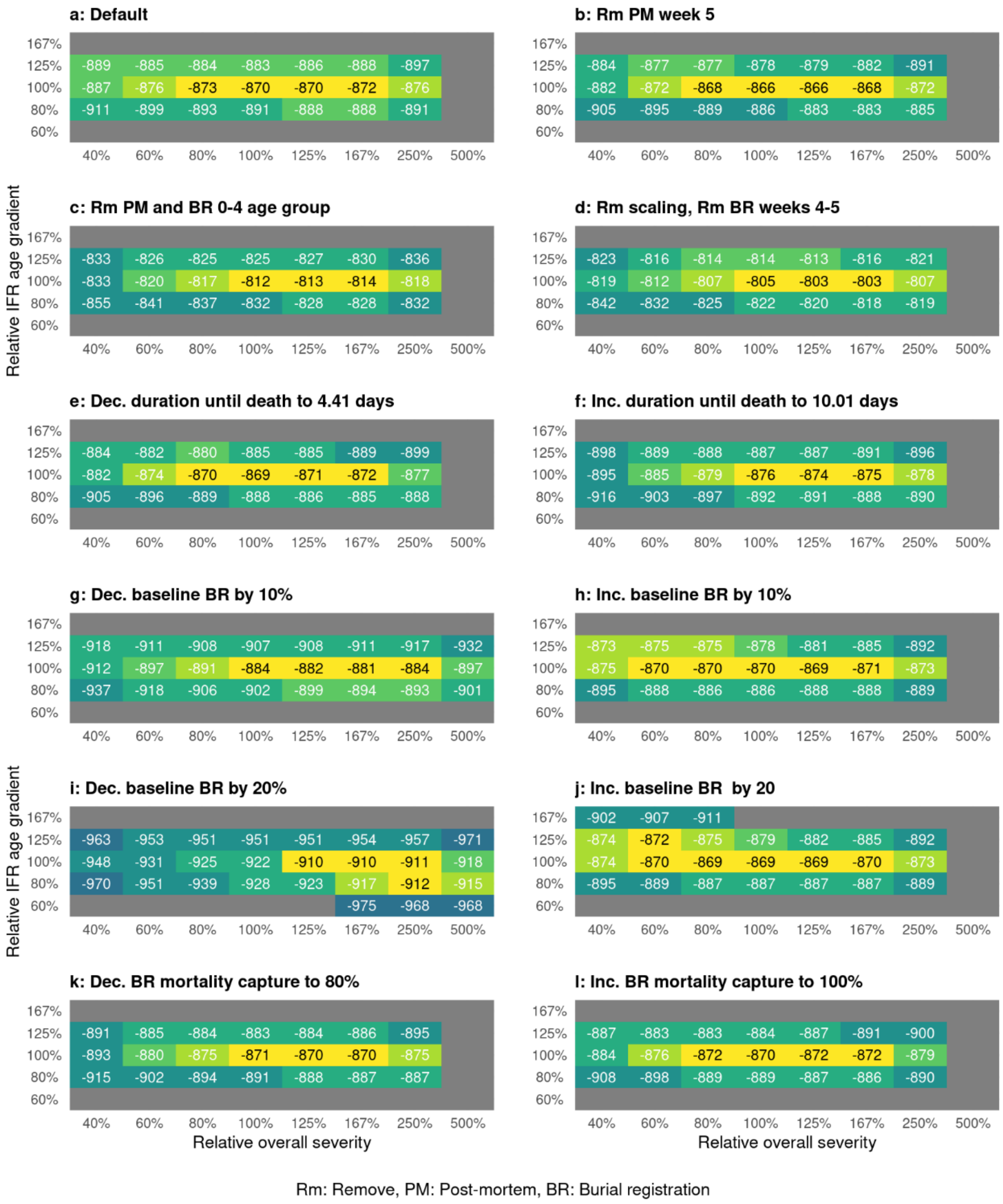

Supplementary Figure 7|Sensitivity analyses on inference of age-gradient and scale of severity. Each heatmap shows the log of average posterior model fits over 100 samples for varied combinations of age gradient and overall infection-fatality ratio (IFR). (a) shows default model assumptions as a point of comparison. (b) Model not fit to post-mortem data during 13th-19th July 2020. (c) Model not fit to burial registry or post-mortem data from 0-4 age group. (d) Model fit to unscaled estimates of excess mortality and not fit to weeks 4-5 of burial registrations. Duration until death following illness that would benefit from hospitalisation decreased to 4.41 days (e) or increased to 10.01 days (f). Relative rate of baseline deaths (relative to 0-4 age group) decreased or increased by 10% (g and h, respectively) or by 20% (i and j, respectively). Burial registration rate capture decreased to 80% (k) or increased to 100% (l).

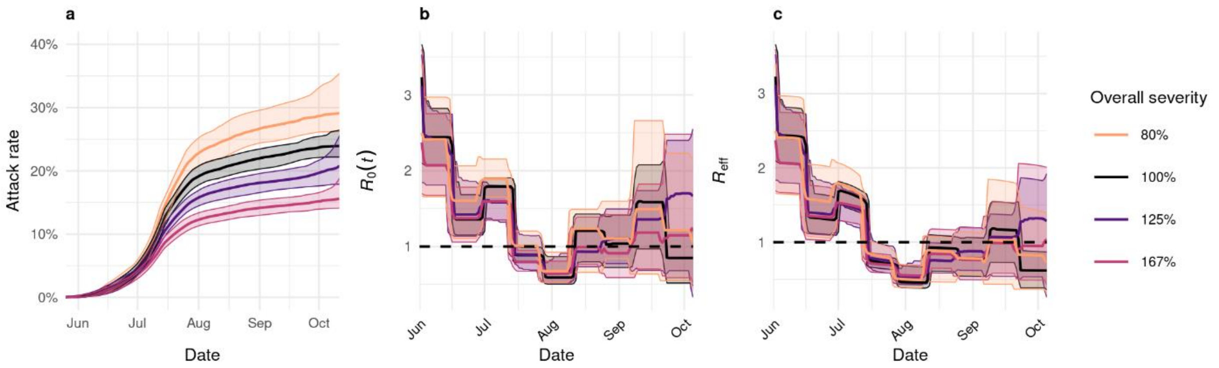

Supplementary Figure 8|Estimates of spread and transmissibility of infection in best-fitting models varying assumptions of IFR age-gradient and overall IFR. Attack rate (a), $R_0(t)$ (b), and R_{eff} (c) trends showing median and 95% credible intervals of 100 samples for each best model fit. Best model fits included an overall IFR from 80-167% (differentiated by colour) with default age-gradient assumptions.

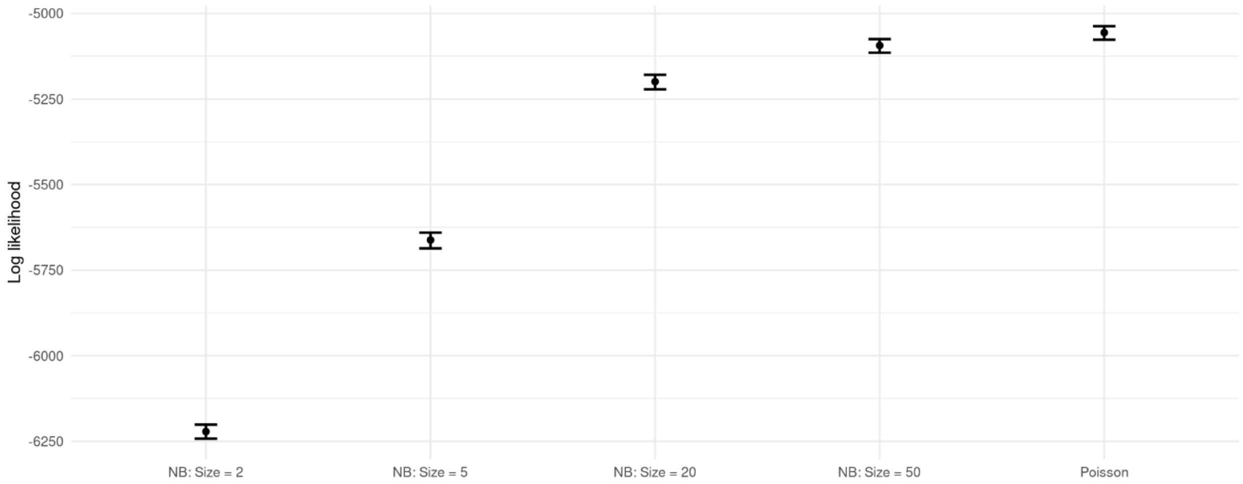

Supplementary Figure 9|Log likelihood of baseline burial registration models under different registration distribution assumptions. Points and bars show median log likelihood and 95% credible intervals for 3,000 draws from the joint posterior of the model fit to burial registration data, assuming burial registrations follow a Poisson or negative binomial distribution with a range of overdispersion assumptions. “Size” in the “dnbinom” R function is inversely correlated with overdispersion such that overdispersion decreases and the negative binomial distribution approaches the Poisson distribution as size increases.

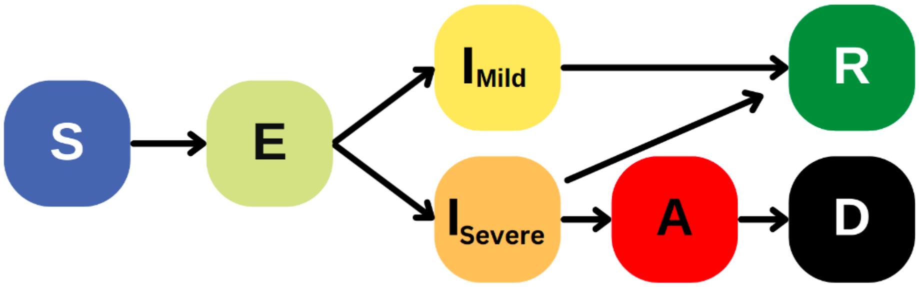

Supplementary Figure 10|Age structured compartmental model structure. Susceptible individuals (S) are infected by SARS-CoV-2 at a rate determined by an age-specific force of infection that utilises given age-specific contact patterns. Infected individuals experience a period of latency (E), followed by disease progression into either mild disease (I_{Mild}, including asymptomatic individuals) or disease sufficiently severe to benefit from hospitalisation (I_{Severe}), regardless of whether they themselves are hospitalised. A proportion of severe disease cases, determined by the selected infection-fatality-ratio (IFR) pattern, then transition to an antemortem stage (A), following which death (D) occurs. Mild disease cases and the remaining severe disease cases that do not result in death result in recovery (R).

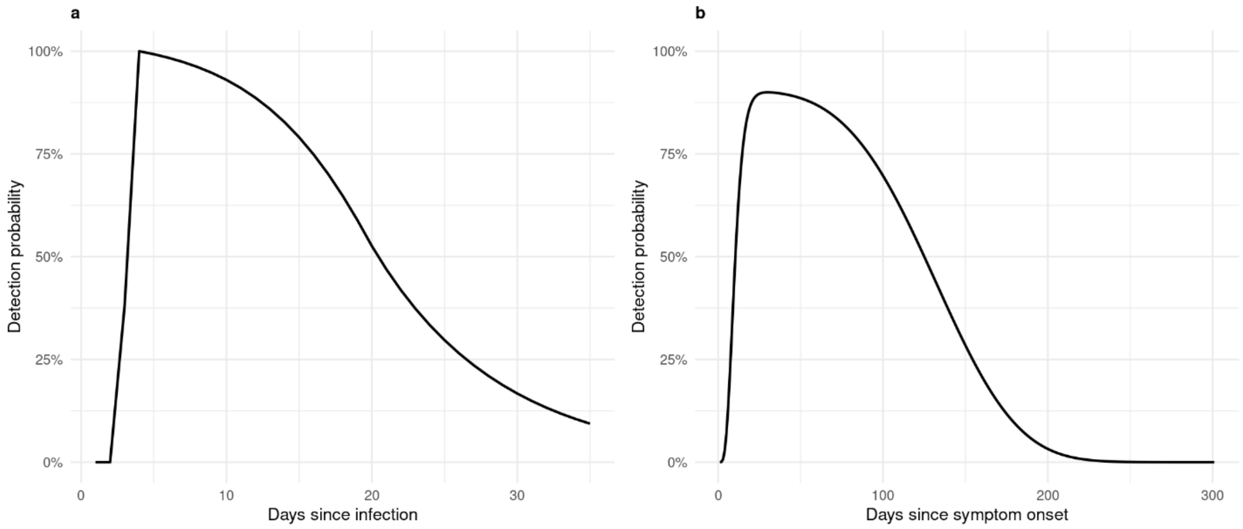

Supplementary Figure 11|Polymerase chain reaction positive (a) and seroprevalence (b) detection probabilities.

Supplementary Tables

Panel	Description	Effects
A	**Default Model Assumptions**	Best fitting envelope (within 4 of best fitting log-likelihood):<br>**80-167% overall severity**
B	**Week 5 of post-mortem data removed from likelihood fitting.**<br><br>Assumptions: Post-mortem prevalence during week 5 is anomalous. 6 tests of which 4 were positive at the CT<40 threshold.<br><br>Potential biases: Removing accurate data points reflecting higher prevalence in week 5.	Best fitting envelope:<br>**80-167% overall severity**<br>No fundamental change.
C	**0-4 age group burial registration and post-mortem data removed from likelihood fitting.**<br><br>Assumptions: The 0-4 age group is affected by processes that increase post-mortem prevalence beyond that reflected in the model.<br><br>Potential biases: Removing accurate data points reflecting higher prevalence in age group 0-4.	Best fitting envelope:<br>**100%-167% overall severity**<br>Small increase in lower bound of best fitting models.
D	**Modelled weekly COVID-19 deaths not scaled using 0-4 age weekly scaling factor based on 2018-2019 median burial registration. Weeks 4-5 of burial registration data removed from the likelihood fitting.**<br><br>Assumptions: Changes in 0-4 age burial registrations *are* reflective of trends in underlying mortality and do not need scaling using age 2018-2019 median. Within this, burial registrations from weeks 4-5 are anomalous.<br><br>Potential biases: Underestimating total mortality based inaccurate representation of changes in burial registrations.	Best fitting envelope:<br>**100%-167% overall severity**<br>Small increase in lower bound of best fitting models.

E-F	**Duration until death for cases of non-hospitalised disease increased or decreased (to match hospitalised disease).**<br><br>Assumptions: Time to death for non-hospitalised disease (BID, assumed to be 70% of the population) is 20% of (4.41 days overall) or equal to (10.01 days) hospitalised disease.<br><br>Potential biases: Time to death over or underestimated in non-hospitalised disease.	Best fitting envelope:<br>**E: 80-167% overall severity**<br>No fundamental change.<br><br>**F: 100-167% overall severity**<br>Small increase in lower bound of best fitting models.
G-J	**Estimates of age 5+ baseline mortality are 10% or 20% lower or higher than pre-pandemic levels.**<br><br>Assumptions: 5+ age group dependence on 0-4 age baseline mortality during 2020 is 10% lower (F), 10% higher (G), 20% lower (H) or 20% higher (I) than pre-pandemic levels.<br><br>Potential biases: Under or overestimation of non-COVID-19 deaths (see main text for examples of what a 10% or 20% shift would be equivalent to)	Best fitting envelope:<br>G: **100-250% overall severity**<br>Small increase in upper and lower bound of best fitting models.<br><br>H: **60-167% overall severity**<br>Small decrease in lower bound of best fitting models.<br><br>I: **125-250% overall severity and 250% overall severity at 80% slope**<br>Increased upper and lower bounds, some improved fits with flatter age-gradient.<br><br>J: **60-167% overall severity and 125% overall severity at 60% slope.**<br>Small decrease in lower bound of best fitting models with improved fits with steeper age-gradient.
K-L	**Modelled COVID-19 deaths not scaled or scaled by 80% to account for incomplete registration.**<br><br>Assumptions: 80% (K) or 100% (L) of mortality is captured in burial registration records (weekly scaling remains as standard).<br><br>Potential biases: Under or overestimation of underlying mortality	K: **100-167% overall severity**<br>Small increase in lower bound.<br><br>**L: 80-167% overall severity**<br>No fundamental change.

Supplementary Table 1: Description of sensitivity analyses performed.

Parameter	Symbol	Value		Description
**Epidemiological parameter**				
Transmission parameter.	β	-		Calculated from R_0.
Basic reproduction number.	R_0	-		Estimated from model fitting.
Mean latent period.	$1/\alpha$	4.6 days.		Estimated at 5.1 days[1]. The last 0.5 days are incorporated in the infectious periods to capture pre-symptomatic infectivity.
Mean duration of mild infection.	$1/\gamma_1$	2.1 days.		Incorporates 0.5 days of infectiousness prior to symptoms. In combination with mean duration of severe illness this gives a mean serial interval of 6.75 days [2].
Mean duration of severe infection.	$1/\gamma_2$	4.5 days.		Mean onset-to-admission of 4 days based on unpublished analysis of data from the ICNARC study[3]. Includes 0.5 days of infectiousness prior to symptom onset.
Mean delay between onset of illness sufficiently severe to benefit from hospitalisation and death.	$1/\gamma_3$	Default: 6.51 days<br>Sensitivity analyses assumes untreated (70%) deaths have durations until death equal to (10.01 days) and 20% of treated deaths (4.41 days).		Calculated based upon squire default parameters using data estimated from unpublished analysis of ICNARC study[3] (see description above).
**Age-stratified parameters**				
Default Age-adjusted IFR.	$\mathrm{IFR}_{\mathrm{Def}}(a)$	0 to 4	0.004	Age-stratified estimates of the IFR from Brazeau et al.[4]
		5 to 9	0.007	
		10 to 14	0.011	
		15 to 19	0.017	
		20 to 24	0.026	
		25 to 29	0.041	
		30 to 34	0.064	
		35 to 39	0.100	
		40 to 44	0.156	
		45 to 49	0.245	
		50 to 54	0.384	
		55 to 59	0.601	
		60 to 64	0.941	
		65 to 69	1.473	
		70 to 74	2.307	

		75 to 79<br>80+	3.613<br>6.914	
Default proportion of infections that require hospitalisation	$\varphi_{2,\mathrm{Def}}(a)$	0 to 4<br>5 to 9<br>10 to 14<br>15 to 19<br>20 to 24<br>25 to 29<br>30 to 34<br>35 to 39<br>40 to 44<br>45 to 49<br>50 to 54<br>55 to 59<br>60 to 64<br>65 to 69<br>70 to 74<br>75 to 79<br>80+	0.001<br>0.001<br>0.001<br>0.002<br>0.003<br>0.05<br>0.007<br>0.009<br>0.013<br>0.018<br>0.025<br>0.036<br>0.050<br>0.071<br>0.100<br>0.140<br>0.233	Smooth scaling age-stratified estimate of the proportion of infections that require hospitalisation from Salje et al.[5]
Simulated proportion of infections that require hospitalisation.	$\varphi_1(a)$	See equations 12-16.		
Simulated Proportion of hospitalised cases dying.	$\varphi_2(a)$	See equations 12-16.		

Supplementary Table 2|Parameter descriptions and values.

Parameter	Symbol	Distribution	Prior	Range
Start Date.	t_0	Uniform.	-	10-55 days relative to the first 10 cumulative deaths.
Basic Reproduction Number.	R_0	Normal.	Mean = 3, sd = 1	[1.6,5.6]
Pseudo-random walk parameters.	ρ_i	Uniform.	-	[-1,1]

Supplementary Table 3|Priors used in squire model fitting.

Supplementary Methods

K-fold cross validation

K-fold cross-validation was applied to our model fitting process to investigate the consistency of age-specific relative rate parameters as registrations in persons aged under 5 years changes, using 26 randomly selected four-week groups of registrations from 2018-2019, (with no replacement). The results of each cross validation are compared with summed burial registrations in each four-week group set for each age group in Supplementary Figure 1.

Mathematical Model of SARS-CoV-2 transmission and disease progression

We modelled the dynamics of a SARS-CoV-2 outbreak using an SEIR model structure ("squire") adapted from that used in Walker et al.[6] and Watson et al.[7]. This model uses estimates of key parameters from 2020 determining the natural history and spread of the virus. The standard model (downloadable as an R package from https://github.com/mrc-ide/squire[8]) is age-stratified and explicitly incorporates mixing patterns across and between different age groups. For these analyses, the deterministic model implementation of squire v0.7.1 was used, with 5 initially infected individuals distributed randomly by age to seed the epidemic.

Following infection, the default implementation of squire is structured in order to capture severity pathways including indication for requiring hospitalisation and intensive care. Given the computational intensity of our analysis, and the observation that in Lusaka a high proportion of mortality occurs outside of hospital[9,10], we developed a simplified framework (see Supplementary Figure 9) that maintains the generation time distribution of the default structure, whilst permitting easier control of the infection-to-death delay distribution (a key uncertainty in the context of mortality occurring outside of the health system). All model states are specified by age group (a) at a given time (t) and include the susceptible population, $S(t, a)$, with the subsequent 5.1 day latent period split across two sequential stages $E_0(t, a)$ and $E_1(t, a)$ in order to generate an Erlang distributed waiting time. Subsequently, individuals follow one of two possible pathway: either entering two sequential states of infection that are either asymptomatic or mildly symptomatic $I_{\text{Mild},0}(t, a)$ and $I_{\text{Mild},1}(t, a)$ or two sequential states for infections that are symptomatic and would subsequently benefit from hospitalisation, $I_{\text{Severe},0}(t, a)$ and $I_{\text{Severe},1}(t, a)$. To ensure the generation time distribution of COVID-19 matches those in the literature to which squire was originally calibrated[2], the durations in these infectious stages, and age-dependent probabilities of severe disease (where mathematically possible given a selected IFR pattern) are maintained to their defaults (Supplementary Table 2). Aside from this factor, we do not rely upon the distinction between severe and mild disease for the remainder of our analysis. Following completion of the infectious period those who will eventually die from the disease pass through two antemortem categories, $A_0(t, a)$ and $A_1(t, a)$, to produce the requisite delay between onset of severe disease and death.

Those that die or recover are denoted by $D(t, a)$ and $R(t, a)$, respectively. Given the relatively short timeframe of analysis, June-October 2020, prior to the emergence of the alpha and beta variants of concern, those that have recovered are deemed effectively immune to reinfection for the remainder of

the simulation. The social contact mixing matrix is given by $c(a, a')$, denoting contacts between individuals in age-groups a and a'.

The differential equations describing the model in full are shown below (Equations 1-11), with the parameter symbols, description and values shown in Supplementary Table 2.

$$\frac{dS(t,a)}{dt} = -\beta \frac{S(t,a)}{N} \sum_{d} c(a,a')[I_{\text{Mild}}(t,a') + I_{\text{Severe}}(t,a')] \tag{1}$$

$$\frac{dE_0(t,a)}{dt} = \beta \frac{S(t,a)}{N} \sum_{d} c(a,a')[I_{\text{Mild}}(t,a') + I_{\text{Severe}}(t,a')] - 2\alpha E_0(t,a) \tag{2}$$

$$\frac{dE_1(t,a)}{dt} = 2\alpha E_0(t,a) - 2\alpha E_1(t,a) \tag{3}$$

$$\frac{dI_{\text{Mild},0}(t,a)}{dt} = (1-\varphi_1(a))2\alpha E_1(t,a) - 2\gamma_1 I_{\text{Mild},0}(t,a) \tag{4}$$

$$\frac{dI_{\text{Mild},1}(t,a)}{dt} = 2\gamma_1 I_{\text{Mild},0}(t,a) - 2\gamma_1 I_{\text{Mild},1}(t,a) \tag{5}$$

$$\frac{dI_{\text{Severe},0}(t,a)}{dt} = \varphi_1(a)2\alpha E_1(t,a) - 2\gamma_2 I_{\text{Severe},0}(t,a) \tag{6}$$

$$\frac{dI_{\text{Severe},1}(t,a)}{dt} = 2\gamma_2 I_{\text{Severe},0}(t,a) - 2\gamma_2 I_{\text{Severe},1}(t,a) \tag{7}$$

$$\frac{dA_0(t,a)}{dt} = \varphi_2(a)2\gamma_2 I_{\text{Severe},1}(t,a) - 2\gamma_3 A_0(t,a) \tag{8}$$

$$\frac{dA_1(t,a)}{dt} = 2\gamma_3 A_0(t,a) - 2\gamma_3 A_1(t,a) \tag{9}$$

$$\frac{dR(t,a)}{dt} = 2\gamma_1 I_{\text{Mild},1}(t,a) + (1-\varphi_2(a))2\gamma_2 I_{\text{Severe},1}(t,a) \tag{10}$$

$$\frac{dD(t,a)}{dt} = 2\gamma_3 A_1(t,a) \tag{11}$$

Probabilities of mortality conditional on severe disease, $\varphi_2(a)$, are derived conditional on the given age-dependent IFR patterns used within the simulation. Default IFR patterns by age estimated as the joint-posterior median of Brazeau et al.[4] are denoted $\text{IFR}_{\text{Def}}(a)$. We then generate alternative assumptions with respect to these IFR patterns by varying the overall IFR within the population according to a multiplier s of the default when adjusted for the demography of Lusaka (pop_w) and the age-gradient of the IFR pattern according to a multiplier g of the default age-specific log IFR curve:

$$\text{IFR}_{\text{Sim}}(a,s,g) = s\overline{\text{IFR}_{\text{Def}}} \frac{\exp[g\log(\text{IFR}_{\text{Def}}(a))]\,\text{pop}_w(a)}{\sum \exp[g\log(\text{IFR}_{\text{Def}}(a))]\,\text{pop}_w(a)}, \tag{12}$$

where

$$\overline{\mathrm{IFR_{Def}}} = \mathrm{IFR_{Def}}(a)\mathrm{pop}_w(a) \tag{13}$$

and

$$\mathrm{pop}_w(a) = \frac{\mathrm{pop}(a)}{\sum \mathrm{pop}(a)}. \tag{14}$$

For our assessment of the relative fit of the different IFR assumptions, s and g were then sampled systematically on the geometric scale. A given IFR pattern ($\mathrm{IFR_{Sim}}$) was then incorporated into the model structure by adjusting $\varphi_2(a)$ parameters, holding the age-dependent probabilities of severe disease $\varphi_1(a)$ to their defaults as derived from Salje et al.[11] (denoted $\varphi_{1,\mathrm{Def}}(a)$) wherever mathematically feasible to maintain the generation time originally derived for squire. For more extreme assumptions, with respect to IFR patterns, age-specific IFR occasionally exceeded $\varphi_{1,\mathrm{Def}}(a)$, necessitating alterations to $\varphi_1(a)$ used within the simulations to generate the required IFR pattern (as specified in equations 15 and 16), however such changes had negligible impact on the average infectious period used within the model with the mean ranging between 2.2-2.4 days.

$$\varphi_2(a) = \min\left(\frac{\mathrm{IFR_{Sim}}(a,s,g)}{\varphi_{1,\mathrm{Def}}(a)}, 1\right) \tag{15}$$

$$\varphi_1(a) = \max\big(\varphi_{2,\mathrm{Def}}(a), \mathrm{IFR_{Sim}}(a,s,g)\big) \tag{16}$$

We also adjusted the delay distribution between the onset of severe disease and death relative to squire defaults to account for the likely more rapid progression of severe disease in those who are not admitted to hospital (found by Mwananyanda et al.[12] to represent 70% of all deaths). Squire's default parameterisation, based upon estimates obtained from data from UK-based patients during the early stages of the pandemic[3,8], produces an average delay between admission to hospital and death of 10.0 days. In the absence of more detailed data for Lusaka we use this mean duration for the 30% of deaths which occurred in hospital and apply a halving in survival time for the 70% brought in dead, producing a weighted delay of 6.5 days. We then conduct a sensitivity analysis with an upper limit of a mean duration of 10.0 days and a lower limit of 4.4 days, representing an assumption of an 80% reduction in survival time in those who would requires hospitalisation but were not able to access to care.

Model fitting

We developed a methodological framework to fit our model to mortality and SARS-CoV-2 prevalence data sources from Lusaka. The model fitting process varies the start date of the pandemic (t_0), the basic reproduction number (R_0) and several pseudo-random walk parameters (ρ_i) introduced at two-week intervals to capture changes in transmission. The time-varying reproduction number (R_t) is calculated in equation 17, where $f(x) = 2e^x/(1+e^x)$**,** following previously used transmission models[13]. Each ρ_i parameter is set to 0 until they sequentially come into effect at two-week intervals.

$$R_0(t) = R_0 f(1 - \rho_1 - \rho_2 \dots \rho_n) \tag{17}$$

A Bayesian framework using a Metropolis-Hastings Markov Chain Monte Carlo (MCMC) based sampling scheme, with the adaptive tuning of the proposal during sampling implementation using the Johnstone-Chang optimisation algorithm[14]. Reported parameter inferences are based on 8 chains of 30,000 iterations, with the first 10,000 discarded as burn-in. Details on prior distributions are given in Supplementary Table 3.

We sampled 100 parameter sets from the MCMC chains weighted by their log likelihood for each model fit. These parameter samples were used to simulate SARS-CoV-2 epidemics in Lusaka, including SARS-CoV-2 prevalence and COVID-19 fatalities that were used in our likelihood functions and then averaged to generate the overall likelihood. The model likelihood was written to match the available data sources, including burial registrations, cross-section population prevalence[15] and post-mortem PCR prevalence[12].

Our Bayesian inferential framework for during the pandemic period can be represented thus:

$$P(B, M, \mathrm{CX} | R_t, t_0, \mathrm{IFR}_{\mathrm{Sim}}(a, s, g), \Theta) \propto \int_{\mathrm{ncd}_{A,T}} \left[P\left(B \middle| \mathrm{ncd}_{a,t}, \mathrm{cd}_{a,t}\right) P\left(M \middle| \mathrm{ncd}_{a,t}, \mathrm{cd}_{a,t}, p_{a,t}\right)\right] \mathrm{d}\, \mathrm{ncd}_{a,t} \, P\left(\mathrm{CX} \middle| p_{a,t}, s_{a,t}\right) P\left(\mathrm{cd}_{a,t}, p_{a,t}, s_{a,t} \middle| R_t, t_0, \mathrm{IFR}_{\mathrm{Sim}}(a, s, g), \Theta\right) P(R_t) P(t_0) P(\mathrm{IFR}_{\mathrm{Sim}}(a, s, g)) P(\Theta) \quad (18)$$

where B, M and CX represent burial registration data, mortuary sampling data and cross-sectional population-based prevalence sampling data respectively. R_t represents the underlying trends in the reproduction number to be fitted within the framework (inclusive of R_0 and all ρ_i parameters), t_0 represents the simulation starting date, $\mathrm{IFR}_{\mathrm{Sim}}(a, s, g)$ represents the patterns of IFR by age (a) for a given IFR slope (s) and gradient (g), Θ represents the remaining model parameters and structural assumptions inherent within the transmission modelling framework. $\mathrm{ncd}_{a,t}, \mathrm{cd}_{a,t}, p_{a,t}$ and $s_{a,t}$ represent, respectively, the number of non-COVID-19 burial registrations, COVID-19 deaths, PCR prevalence and seroprevalence by age (a) and time (t).

The framework can then be viewed as being comprised of observation models for the burial registrations data, $P\left(B \middle| \mathrm{ncd}_{a,t}, \mathrm{cd}_{a,t}\right)$, the mortuary data $P\left(M \middle| \mathrm{ncd}_{a,t}, \mathrm{cd}_{a,t}, p_{a,t}\right)$ and the cross-sectional survey $P\left(\mathrm{CX} \middle| p_{a,t}, s_{a,t}\right)$, with uncertainty in the number of non-COVID-19 deaths by age integrated over by drawing 1000 samples of our model fitted to pre-COVID-19 mortality patterns (See section *Methods: Modelling SARS-CoV-2 transmission*). $P\left(\mathrm{cd}_{a,t}, p_{a,t}, s_{a,t} \middle| R_t, t_0, \mathrm{IFR}_{\mathrm{Sim}}(a, s, g), \Theta\right)$ represents the underlying transmission model, with $P(R_t)$ and $P(t_0)$ representing priors described in Supplementary Table 1, whereas $\mathrm{IFR}_{\mathrm{Sim}}(a, s, g)$ and Θ are held as constant within each individual fit, with parameters determining $\mathrm{IFR}_{\mathrm{Sim}}(a, s, g)$ grid-sampled and Θ held to default values with the exception of sensitivity analyses as outlined in *Results: SARS-CoV-2 transmission and COVID-19 severity during the first wave*.

Burial registration data observational model – $P\left(B \middle| \mathrm{ncd}_{a,t}, \mathrm{cd}_{a,t},\right)$

We fitted the model to the weekly age-grouped burial registrations, $B = \{B_{a,t}\}$, during the 16 weeks of the post-mortem study (15th June-October 4th). To take into account variation in overall registration patterns, a weekly scaling factor (sf_t, the ratio of median burial registrations of children aged 0-4 in 2018-2019 to weekly non-COVID-19 deaths in children aged 0-4 in the study period) was applied to COVID-19 deaths output by the model, which was then added to baseline estimates of burial

registration (see *Results: Estimating excess mortality in Lusaka during 2020 to mid-2021* and *Methods: Estimating excess mortality in Lusaka using burial registration data*) to generate total modelled deaths at the burial registry level.

$$B_{a,t}|\mathrm{ncd}_{a,t}, \mathrm{cd}_{a,t} \sim \mathrm{Pois}\left(\mathrm{ncd}_{a,t} + \mathrm{cd}_{a,t} \cdot \mathrm{sf}_t\right), \tag{19}$$

where

$$\mathrm{sf}_t = 0.9 \times \frac{\mathrm{Mean}\left(B_{1,2018/19}\right)}{\mathrm{ncd}_{1,t}}. \tag{20}$$

Mortuary data observational model – $P\left(M|\mathrm{ncd}_{a,t}, \mathrm{cd}_{a,t}, p_{a,t}\right)$

The model was simultaneously to the weekly age-grouped post-mortem test data, $M = \left\{M_{a,t}^{\mathrm{positive}}, M_{a,t}^{\mathrm{total}}\right\}$, during the 16 weeks of the study (CT<40). We calculated SARS-CoV-2 PCR prevalence in the mortuary by dividing the sum of modelled COVID-19 deaths in the mortuary ($\mathrm{cd}_{a,t}$, with scaling factor) and non-causal COVID-19 deaths by the sum of modelled COVID-19 deaths ($\mathrm{cd}_{a,t}$, with scaling factor) and non-COVID-19 deaths ($\mathrm{ncd}_{a,t}$). Non-causal COVID-19 deaths were the product of non-COVID-19 deaths ($\mathrm{ncd}_{a,t}$) generated from our excess mortality analysis with the population PCR prevalence (*p*), that we took from the mid-point of each week, Thursday).

$$M_{a,t}^{\mathrm{positive}}|\mathrm{ncd}_{a,t}, \mathrm{cd}_{a,t}, p_{a,t} \sim \mathrm{Bin}\left(M_{a,t}^{\mathrm{total}}, \frac{\mathrm{cd}_{a,t} \cdot \mathrm{sf}_t + \mathrm{ncd}_{a,t} p_{a,t}}{\mathrm{cd}_{a,t} \cdot \mathrm{sf}_t + \mathrm{ncd}_{a,t}}\right) \tag{21}$$

The probability of a positive PCR test given infection was sourced from data published by Hay et al.[16]. While our code allows scaling of sensitivity, our default assumptions are that the maximum sensitivity is 100% which occurs at 4 days following infection (Supplementary Figure 10).

Cross-sectional population prevalence survey observational model – $P\left(\mathrm{CX}|p_{a,t}, s_{a,t}\right)$

We used the probability of testing positive by PCR as a function since time from infection as estimate by Hay et al. [16] and the probability of testing positive by serology as a function of time since symptom onset as estimated by Brazeau et al. [17], which accounts for both seroconversion and seroreversion[18] (see Supplementary Figure 10).

Both PCR and serological prevalence were then averaged across age groups throughout the survey period in Lusaka, $\tau =$ 4th-19th July 2020, and the likelihood of the observed data, $\mathrm{CX} = \left\{p_{\mathrm{positive}}, p_{\mathrm{samples}}, s_{\mathrm{positive}}, s_{\mathrm{samples}}\right\}$ are then calculated according to the following binomial distributions:

$$p_{\mathrm{positive}} \sim \mathrm{Bin}\left(p_{\mathrm{samples}}, \overline{p_\tau}\right) \tag{22}$$

$$s_{\mathrm{positive}} \sim \mathrm{Bin}\left(s_{\mathrm{samples}}, \overline{s_\tau}\right) \tag{23}$$

Supplementary References

1. Lauer, S. A. *et al.* The incubation period of coronavirus disease 2019 (CoVID-19) from publicly reported confirmed cases: Estimation and application. *Ann Intern Med* **172**, 577–582 (2020).

2. Bi, Q. *et al.* Epidemiology and transmission of COVID-19 in 391 cases and 1286 of their close contacts in Shenzhen, China: a retrospective cohort study. *Lancet Infect Dis* **20**, 911–919 (2020).

3. Intensive Care National Audit & Research Centre (ICNARC). *ICNARC report on COVID- 19 in critical care 10 April 2020*. www.icnarc.org/About/Latest- News/2020/04/04/Report-On-2249-Patients-Critically-Ill-With-Covid-19 (2020).

4. Brazeau, N. F. *et al.* Estimating the COVID-19 infection fatality ratio accounting for seroreversion using statistical modelling. *Communications Medicine* **2**, 1–13 (2022).

5. Salje, H. *et al.* Estimating the burden of SARS-CoV-2 in France. *Science (1979)* **369**, 208–211 (2020).

6. Walker, P. G. T. *et al.* The impact of COVID-19 and strategies for mitigation and suppression in low- And middle-income countries. *Science (1979)* **369**, 413–422 (2020).

7. Watson, O. J. *et al.* Leveraging community mortality indicators to infer COVID-19 mortality and transmission dynamics in Damascus, Syria. *Nat Commun* **12**, 1–10 (2021).

8. Watson, O. J., Walker, P., Whittaker, C., Winskill, P. & Charles, G. SEIR transmission model of COVID-19 • squire. https://mrc-ide.github.io/squire/ (2022).

9. Yokobori, Y. *et al.* Analysis of causes of death among brought-in-dead cases in a third-level Hospital in Lusaka, Republic of Zambia, using the tariff method 2.0 for verbal autopsy: A cross-sectional study. *BMC Public Health* **20**, 1–9 (2020).

10. Lapidot, R. *et al.* Verbal autopsies for out-of-hospital infant deaths in Zambia. *Pediatrics* **147**, (2021).

11. Salje, J., Gayathri, P. & Löwe, J. The ParMRC system: molecular mechanisms of plasmid segregation by actin-like filaments. *Nat Rev Microbiol* **8**, 683–692 (2010).

12. Mwananyanda, L. *et al.* Covid-19 deaths in Africa: Prospective systematic postmortem surveillance study. *The BMJ* **372**, 9–13 (2021).

13. Unwin, H. J. T. *et al.* State-level tracking of COVID-19 in the United States. *Nat Commun* **11**, (2020).

14. Johnstone, R. H. *et al.* Uncertainty and variability in models of the cardiac action potential: Can we build trustworthy models? *J Mol Cell Cardiol* **96**, 49–62 (2016).

15. Mulenga, L. B. *et al.* Prevalence of SARS-CoV-2 in six districts in Zambia in July, 2020: a cross-sectional cluster sample survey. *Lancet Glob Health* **9**, e773–e781 (2021).

16. Hay, J. A. *et al.* Estimating epidemiologic dynamics from cross-sectional viral load distributions. *Science (1979)* **373**, (2021).

17. Brazeau, N. F. *et al.* Report 34 - COVID-19 infection fatality ratio estimates from seroprevalence. *Imperial College London* 1–18 (2020).

18. Muecksch, F. *et al.* Longitudinal analysis of clinical serology assay performance and neutralising antibody levels in COVID19 convalescents. *medRxiv* 1–22 (2020) doi:10.1101/2020.08.05.20169128.
