# Supplementary material for: Using mortuary and burial data to place COVID-19 in Lusaka, Zambia within a global context
Source: Nat Commun. 2023 Jun 29;14:3840. doi: 10.1038/s41467-023-39288-6 (PMC10307769; doi:10.1038/s41467-023-39288-6)
Supplement: Supplementary file 3 — Reporting Summary [file 41467_2023_39288_MOESM3_ESM.pdf]

nature portfolio

Corresponding author(s): Dr Patrick Walker

Last updated by author(s): May 23, 2023

Reporting Summary

Nature Portfolio wishes to improve the reproducibility of the work that we publish. This form provides structure for consistency and transparency in reporting. For further information on Nature Portfolio policies, see our Editorial Policies and the Editorial Policy Checklist.

Statistics

For all statistical analyses, confirm that the following items are present in the figure legend, table legend, main text, or Methods section.

n/a	Confirmed	
☐	☒	The exact sample size (*n*) for each experimental group/condition, given as a discrete number and unit of measurement
☒	☐	A statement on whether measurements were taken from distinct samples or whether the same sample was measured repeatedly
☒	☐	The statistical test(s) used AND whether they are one- or two-sided<br>*Only common tests should be described solely by name; describe more complex techniques in the Methods section.*
☒	☐	A description of all covariates tested
☒	☐	A description of any assumptions or corrections, such as tests of normality and adjustment for multiple comparisons
☐	☒	A full description of the statistical parameters including central tendency (e.g. means) or other basic estimates (e.g. regression coefficient) AND variation (e.g. standard deviation) or associated estimates of uncertainty (e.g. confidence intervals)
☒	☐	For null hypothesis testing, the test statistic (e.g. *F*, *t*, *r*) with confidence intervals, effect sizes, degrees of freedom and *P* value noted<br>*Give P values as exact values whenever suitable.*
☐	☒	For Bayesian analysis, information on the choice of priors and Markov chain Monte Carlo settings
☒	☐	For hierarchical and complex designs, identification of the appropriate level for tests and full reporting of outcomes
☒	☐	Estimates of effect sizes (e.g. Cohen's *d*, Pearson's *r*), indicating how they were calculated

Our web collection on statistics for biologists contains articles on many of the points above.

Software and code

Policy information about availability of computer code

Data collection	No software was used in the data collection process.
Data analysis	The data analysis was conducted in R (version 4.2.3), using the drjacoby (version 1.5.2) and squire (version 0.7.1) packages. Source code for the mathematical models developed and used in these analyses is available open access and can be found at: https://github.com/RJSheppard/COVID.IFR.Lusaka.

For manuscripts utilizing custom algorithms or software that are central to the research but not yet described in published literature, software must be made available to editors and reviewers. We strongly encourage code deposition in a community repository (e.g. GitHub). See the Nature Portfolio guidelines for submitting code & software for further information.

Data

Policy information about availability of data

All manuscripts must include a data availability statement. This statement should provide the following information, where applicable:

- Accession codes, unique identifiers, or web links for publicly available datasets
- A description of any restrictions on data availability
- For clinical datasets or third party data, please ensure that the statement adheres to our policy

The WHO excess mortality data and UN World Population Prospects demographic data that support the findings of this study are available from https://www.who.int/data/sets/global-excess-deaths-associated-with-covid-19-modelled-estimates and https://population.un.org/Dataportal/ (accessed 11th May 2022),

respectively, and may be found in the repository (https://github.com/RJSheppard/COVID.IFR.Lusaka) along with the IFR estimates used to generate Figure 2, the Nyanga contact matrix and aggregated (by week and age group) versions of the burial registration, post-mortem prevalence, and population survey datasets. Full burial registration and post-mortem prevalence data may be shared in full, on the basis of a request through a formal data sharing agreement between relevant authors. Deidentified population survey participant data used for this analysis can be requested from the Zambian Ministry of Health, along with the unpublished demography estimates for Lusaka used in this study (interested researchers must submit a research proposal for consideration by the original study investigators. If approved, the requestor must sign a data use agreement). All data requests should be directed to the corresponding author who will facilitate the initiation of relevant processes.

Research involving human participants, their data, or biological material

Policy information about studies with human participants or human data. See also policy information about sex, gender (identity/presentation), and sexual orientation and race, ethnicity and racism.

Reporting on sex and gender	The majority of data here come from published sources that investigated sex-based patterns. The population PCR prevalence and seroprevalence surveys sampled equal numbers of males and females, finding no significant difference due to sex. Males were over-represented in the burial registration data (2018-June 2021) in a 57:43 ratio (males=32780, females=24969), though this is to be expected given higher mortality rates in males in Zambia (See demographic and health survey 2018: https://dhsprogram.com/pubs/pdf/FR361/FR361.pdf). In the post-mortem PCR prevalence data, Mwananyanda et al. patterns of mortality by recorded gender in trends by age relative to 2018-19 (Supplementary Figure 1) which supports our assumptions that COVID-19 mortality and non-COVID-19 mortality were not substantially affected by sex. In these datasets collection, it is unknown whether the sex described is based on self-reporting or assignment.
Reporting on race, ethnicity, or other socially relevant groupings	Race and ethnicity do not play a role in the study methodology.
Population characteristics	The population data were grouped by age, due to the age-specific COVID-19 severity trends and age-structured population mixing. Public estimates for population structure in Lusaka can be found at https://www.zamstats.gov.zm/#
Recruitment	Participants for the ZPRIME and COVID-19 expansion studies that generated post-mortem PCR prevalence data were enrolled at the University Teaching Hospital Morgue, with the only exclusion criteria of enrollment more than 48 hours after death to reduce false negatives. The total volume of deaths exceeded the capacity for enrollment, which varied over the study between one in five to one in two deaths.<br><br>Participants for the population level SARS-CoV-2 prevalence study were selected from 16 randomly selected, proportional to size, standard enumeration areas in Lusaka, from which 20 households were selected from all listed households. All individuals who had slept in a household the previous night were eligible for the study.
Ethics oversight	Ethical oversight for ZPRIME and the COVID-19 expansion that generated post-mortem PCR prevalence data from UTH were provided by the institutional review boards at Boston RESEARCH University and the University of Zambia. Written informed consent was obtained from the deceased's family members or representatives.<br><br>The population level SARS-CoV-2 prevalence study was approved by the Zambia National Health Research Authority and the University of Zambia Biomedical Research Ethics Committee. The study was reviewed in accordance with the Centers for Disease Control and Prevention (CDC) human research protection procedures and was determined to be non-research. Written informed consent was obtained for adults (aged ≥18 years) and emancipated minors, parental consent was obtained for participants aged 17 years and younger, and assent was obtained for participants aged 7-17 years, before the study.<br><br>The novel analyses presented here were granted approval via Imperial's Research Governance Integrity framework on the basis of the above pre-existing ethics approvals.

Note that full information on the approval of the study protocol must also be provided in the manuscript.

Field-specific reporting

Please select the one below that is the best fit for your research. If you are not sure, read the appropriate sections before making your selection.

☒ Life sciences ☐ Behavioural & social sciences ☐ Ecological, evolutionary & environmental sciences

For a reference copy of the document with all sections, see nature.com/documents/nr-reporting-summary-flat.pdf

Life sciences study design

All studies must disclose on these points even when the disclosure is negative.

Sample size	This study is a secondary analysis, based on previous collected data. No sample size calculations or decisions were made in this study. The primary datasets used in this study (burial registrations, post-mortem PCR prevalence, population seroprevalence and PCR prevalence) provided a basis from which we could establish the impact of the COVID-19 pandemic on mortality and disease spread, enabling inference on COVID-19 severity.
Data exclusions	Data was not excluded unless specified (i.e., in sensitivity analyses).

Replication	Full details of the model fitting procedue are supplied within the methods and supplementary information, with all necessary code located in an open-access repository https://github.com/RJSheppard/COVID.IFR.Lusaka. The analysis was performed using either 5 (for baseline mortality estimates) or 8 (for transmission model analysis) MCMC chains with which to generate results. A subset of the analysis was performed to assess model fit consistency, finding high consistency across model fits.
Randomization	Data were not collected in these secondary analyses of data from previously published work. Randomization in data collection methods, therefore, was not a necessary part of the analyses, outside of the randomization used in the methodology used for previously collected data (e.g., randomization in population PCR prevalence and seroprevalence).
Blinding	Data were not collected in these secondary analyses of data from previously published work. Blinding in data collection methods, therefore, was not a necessary part of the analyses.

Reporting for specific materials, systems and methods

We require information from authors about some types of materials, experimental systems and methods used in many studies. Here, indicate whether each material, system or method listed is relevant to your study. If you are not sure if a list item applies to your research, read the appropriate section before selecting a response.

Materials & experimental systems

n/a	Involved in the study	
☒	☐	Antibodies
☒	☐	Eukaryotic cell lines
☒	☐	Palaeontology and archaeology
☒	☐	Animals and other organisms
☒	☐	Clinical data
☒	☐	Dual use research of concern
☒	☐	Plants

Methods

n/a	Involved in the study	
☒	☐	ChIP-seq
☒	☐	Flow cytometry
☒	☐	MRI-based neuroimaging
